# Making Succinate More Successful

**DOI:** 10.1289/ehp.113-a832

**Published:** 2005-12

**Authors:** Carol Potera

What does the word “fermentation” bring to mind? Beer? Bread? Ethanol derived from corn and other plant matter? How about succinate? Since 2001, biochemist George Bennett and bioengineer Ka-Yiu San, both professors at Rice University, have been tinkering with *Escherichia coli* to coax it to convert sugars to succinate, a chemical with multiple industrial uses. Now their efforts are bearing fruit as “green” succinate is starting to become a reality in chemical commerce.

Who uses succinate? By itself, succinate is used as a flavor enhancer in food products and as a stabilizer in pharmaceuticals. It is also used to produce other industrial chemicals, including butanediol, tetrahydrofuran, and pyrrolidone, which become ingredients in solvents, paints, deicers, plastics, fuel additives, fabrics, and carpets.

Succinate is traditionally manufactured from petrochemicals through expensive processes. The Rice team’s goal is to make a more environmentally friendly succinate from renewable starting materials. “We want to use agricultural materials that are renewable to make this useful product, and alleviate the drain of limited oil reserves,” says Bennett.

The Department of Energy (DOE) “sees a future for biorefineries that use biomass as feedstocks to make fuels and chemicals,” says department chemist Gene Petersen. In 1994, the agency’s now-defunct Alternative Feedstocks Program assessed the likelihood of making chemicals from biomass. “The category of compounds that seemed most viable were organic acids like succinic, acetic, and citric,” says Petersen.

That evaluation resulted in the DOE’s funding of fermentation research programs at national laboratories and universities. In 2004, the DOE released volume I of a report titled *Top Value Added Chemicals from Biomass*, coauthored by Petersen (volume II is expected out in 2006). According to the report, succinate tops the list of 12 “building block” chemicals—molecules with multiple functional groups that possess the potential to be transformed into new families of useful materials—that can be produced from sugars via biological conversion.

In 2001, 10 million pounds of succinate were produced from petrochemicals and sold for an average of $2 per pound. “The market is there if we can make succinate more economically through biofermentation,” says Praveen Vadlani, principal research scientist at AgRenew Incorporated in Manhattan, Kansas. By making green succinate in bulk—a potentially cheaper material with the cachet of environmental friendliness—people may even be inspired to find new applications for it, such as bio-based polymers and composites, predicts Vadlani.

## Optimizing Glucose

“It’s not a direct route from glucose to succinate,” says Bennett. Several biochemical pathways can produce succinate from sugar. They all start with the degradation of glucose, which contains six carbon atoms, to pyruvate, which contains three carbons. Then pyruvate can be converted not only into succinate (which contains four carbons), but also lactate, ethanol, acetate, and other chemicals. The trick is to speed up the chemical reactions that lead to succinate production while blocking those that make lactate, ethanol, and other chemicals.

Some pathways operate aerobically (they need oxygen) whereas others run anaerobically (they do not use oxygen). Bennett and San spent four years working out both aerobic and anaerobic methods for *E. coli* to convert glucose into almost pure succinate in yields high enough to be commercially feasible. Their anaerobic method has proven more efficient, with 1.0 gram of glucose yielding 1.44 grams of sodium succinate. Their aerobic process yields about three-quarters that amount.

Bennett and San have engineered a form of *E. coli*, dubbed SBS550MG, that contains six genetic alterations that allow it to produce succinate anaerobically from two different routes—the glyoxylate pathway and the fermentation route. To accomplish this, the researchers deleted four *E. coli* genes, including those for lactate and ethanol production, and activated the glyoxylate pathway in order to speed the conversion of glucose solely into succinate. They also added two genes from other bacteria to boost the amount of succinate generated.

Both routes produce succinate through different biochemical reactions that do not compete or interfere with each another. In fact, Bennett and San designed the routes to be complementary. SBS550MG converts glucose to succinate very efficiently and very rapidly, and gives high yields of nearly pure succinate with few by-products, says San. High-pressure liquid chromatography confirms that more than 90% of the starting glucose ends up as succinate.

To make the leap from the laboratory to the marketplace, the Rice scientists teamed up with bioengineering experts at AgRenew. Under Vadlani’s direction, AgRenew will perfect the methods to manufacture succinate from corn and sorghum rather than from the pure glucose used in the laboratory experiments. “We see great promise in the technology, and once the methods are established, we may even switch to cornstalks or agricultural waste,” says Vadlani.

## Up and Running

Kris Berglund, chief science officer at Diversified Natural Products (DNP) in Scottville, Michigan, is experiencing new market demands for green succinate. DNP also uses *E. coli* to ferment sugars to succinate, but the bacterial strain used was licensed from the DOE, which produced it under its Alternative Feedstocks Program. DNP’s fermentation method differs from that created by Bennett and San in that an aerobic process occurs first, followed by an anaerobic process that requires added carbon dioxide. Says Berglund, “We take six carbons from glucose and add two carbons from carbon dioxide to form two molecules of succinate with four carbons each.”

DNP just started large-scale production of succinate from agricultural materials at Agro-Industrie Recherches et Développements (ARD) in Pomacle, France. The joint venture was announced by French president Jacques Chirac on 30 August 2005. In seeking a partner to manufacture its biosuccinate batches, Berglund searched worldwide and chose ARD because “they shared the same vision as we do to replace petroleum-based chemicals with biomass production,” he says.

The staff at ARD’s manufacturing facility, located in the agricultural Champagne region, will produce up to 200 tons of succinate from wheat and sugar beets in the first year. DNP plans to construct a large plant in the United States, too. “As far as we know, we’re the first company to enter commercial production of succinate from biomaterials,” says Berglund. Although production has just begun, Berglund says “customers already want to buy it,” particularly for use as a flavor enhancer, stabilizer, and acidulant for food production. Some customers desire green succinate because they view it as a “natural” ingredient that would be favored by organic food consumers.

Customers also are lining up to buy DNP’s succinate-based runway and wing deicer. Succinate, which lowers the freezing point of water, replaces the formates and acetates in deicers now on the market. These chemicals not only corrode the metal alloy, plastic, and rubber parts of airplanes, but also destroy the concrete surfaces and plastic and metal components of lighting equipment at airports. Federal Aviation Administration approval of the DNP deicer appears imminent, according to Berglund. Other products in the succinate pipeline at DNP include biodegradable solvents that do not cause air pollution or damage the ozone, a diesel fuel additive to reduce particulate emissions, and biodegradable polyesters for use in fabrics or plastics.

DNP does not disclose information about its yields, but “our methods are good enough to compete with any fossil fuel–related process,” says Berglund. Based on estimates calculated when oil sold at $25 per barrel, DNP forecast a selling price of less than $1 a pound for its biosuccinate. With declining petroleum reserves and rising oil prices, “the economics of our process are even more attractive,” says Berglund.

Other companies are following behind on the same commercialization path. Michigan Biotechnology Institute (MBI) International in Lansing developed a patented process based on *Actinobacillus succinogenes*, a bacterium isolated from the cow’s rumen (a fermentation chamber in the animal’s stomach). MBI scientists created mutant strains for anaerobic production of succinate from biomass sugars, resulting in yields of approximately 1 gram of succinate from 1 gram of glucose. Different types of biomass, including cornstalks, corn fiber, and sugarcane, can be used to fuel the fermentation.

The MBI method also pipes in carbon dioxide. “It’s a greenhouse-friendly fermentation, because we utilize carbon dioxide instead of generating carbon dioxide,” says microbiologist Bernie Steele, manager of quality assurance at MBI. He foresees his company’s biosuccinate method being linked to ethanol plants, which generate carbon dioxide as a waste product. An overall biorefinery program that uses by-products from one production stream to feed another manufacturing process maximizes economic returns.

After 10 years of research and development efforts, MBI is seeking partners to scale up its process to manufacture large quantities of green succinate. “The technology is maturing for the transition of biomass into energy or chemicals,” says Steele.

## Future Uses for Succinate

The future for succinate lies not in utilizing it directly as a food additive, but in creating innovative biopolymers like polybutylene succinate. This biodegradable plastic, already being made with petroleum-based succinate, is found in packaging film, bags, flushable hygienic products, and garden mulch. “We have customers waiting to buy our succinate to make polymers,” says Berglund. Other, stiffer biodegradable plastics, like polylactic acid, are formed into drinking cups, food trays, containers, and planter boxes. These “green” alternatives replace products typically made from petroleum-based plastics.

The commitment of corporate giants like Cargill and DuPont to make products from biomass casts “a bright light on the future of biofermentation,” says Petersen. Cargill produces up to 300 million pounds of polylactic acid, sold as NatureWorks→ PLA, from renewable resources such as corn. DuPont’s Sorona→, a polymer of 1,3-propanediol now made from petrochemicals, adds softness and stretch to fabrics. In 2006, DuPont will switch to a fermentation method to make its 1,3-propanediol from corn sugar. Called Bio-PDO∀, the corn-based polymer will be the first product developed by DuPont’s Bio-Based Materials unit.

Despite this buy-in, the future isn’t here yet. In general, the long journey to find an economic way to convert renewable bio-materials into commodity chemicals takes about 10 years; the basic research behind NatureWorks PLA started in the 1980s. “It’s not that easy to get away from petrochemicals, even though we want to environmentally,” says Petersen.

But the large-scale processes under way at Cargill and DuPont indicate long-term business interest in fermentation, says Bennett. He envisions more companies entering the bioproducts business and the economics of succinate and other bioproducts improving through engineering refinements. And as oil prices rise and fermentation becomes more economically appealing, “companies will find different ways to make the same end product,” says Vadlani.

## Figures and Tables

**Figure:**
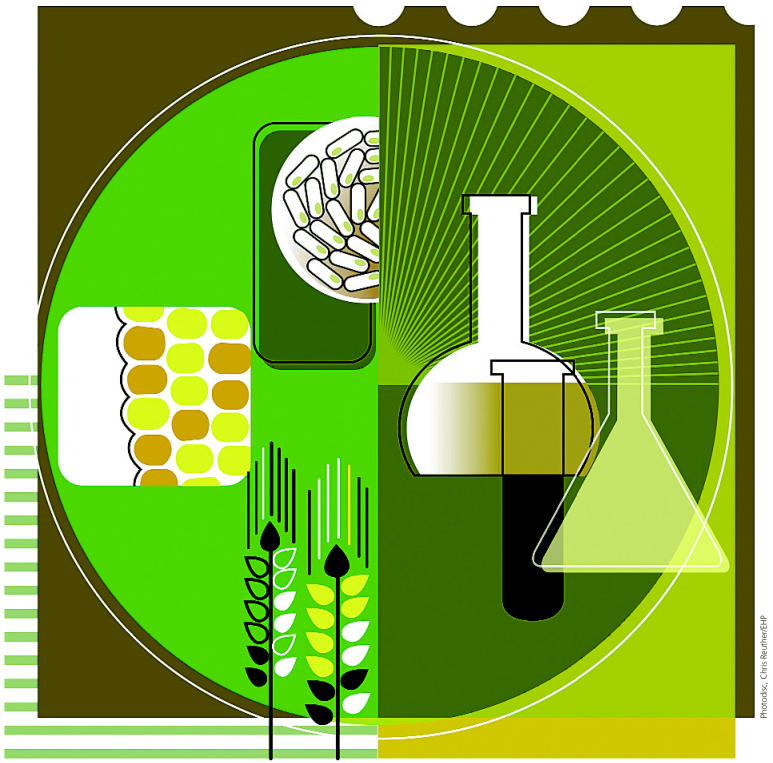


**Figure:**
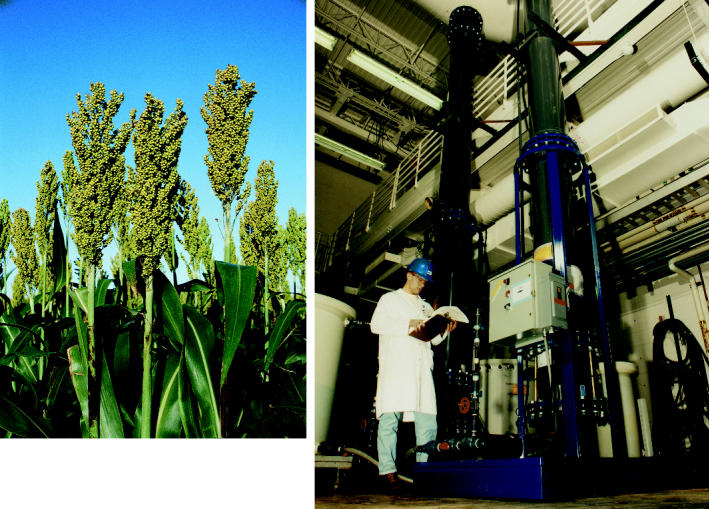
Sweetening the deal Researchers are refining techniques for producing succinate from biomass such as sorghum (above) rather than petroleum. One group, MBI International, uses ion exchange to further refine succinate into succinic acid (right).
